# Development and Evaluation of a Novel One-Step RT-qPCR Targeting the Vero Gene for the Identification of False-Positive Results Caused by Inactivated Virus Vaccine Contamination

**DOI:** 10.3390/vaccines11020372

**Published:** 2023-02-06

**Authors:** Xin-Qi Zheng, Wan-Bao Yang, Lin Xie, Zi-Han Wei, Jiang-Xing Zhuang, Tian-Ci Yang

**Affiliations:** 1 Center of Clinical Laboratory, Zhongshan Hospital of Xiamen University, School of Medicine, Xiamen University, Xiamen 361004, China; 2 Fujian Provincial Key Laboratory of Neurodegenerative Disease and Aging Research, Institute of Neuroscience, School of Medicine, Xiamen University, Xiamen 361102, China; 3 Institute of Infectious Disease, School of Medicine, Xiamen University, Xiamen 361004, China

**Keywords:** SARS-CoV-2, Vero gene, RT-qPCR, inactivated virus vaccine, false positive

## Abstract

To identify false-positive SARS-CoV-2 test results caused by novel coronavirus inactivated vaccine contamination, a novel RT-qPCR targeting the ORF1ab and N genes of SARS-CoV-2 and Vero gene was developed. The amplification efficiency, precision, and lower limit of detection (LLOD) of the RT-qPCR assay were determined. A total of 346 clinical samples and 132 environmental samples were assessed, and the diagnostic performance was evaluated. The results showed that the amplification efficiency of the ORF1ab, N, and Vero genes was 95%, 97%, and 93%, respectively. The coefficients of variation of Ct values at a concentration of 3 × 10^4^ copies/mL were lower than 5%. The LLOD for the ORF1ab, N, and Vero genes reached 8.0, 3.3, and 8.2 copies/reaction, respectively. For the 346 clinical samples, our RT-qPCR assay identified SARS-CoV-2-positive and SARS-CoV-2-negative samples with a sensitivity of 100.00% and a specificity of 99.30% and novel coronavirus inactivated vaccine-contaminated samples with a sensitivity of 100% and a specificity of 100%. For the environmental samples, our RT-qPCR assay identified novel coronavirus inactivated vaccine-contaminated samples with a sensitivity of 88.06% and a specificity of 95.38%. In conclusion, the RT-qPCR assay we established can be used to diagnose COVID-19 and, to a certain extent, false-positive results due to vaccine contamination.

## 1. Introduction

COVID-19 caused by SARS-CoV-2 poses a great burden and challenge to the global public health system due to an extremely high viral infection rate [1,2]. Although some drugs for the treatment of COVID-19 have been approved [3,4], vaccination is considered the most cost-effective intervention to eventually end the COVID-19 pandemic [5]. Molecular detection of SARS-CoV-2 nucleic acid is the gold standard for the diagnosis of COVID-19 [2], but vaccine nucleic acid contamination presents a challenge to this strategy. One study reported the positive detection of SARS-CoV-2 nucleic acid on haemostatic stickers placed on the surface of recipients’ hands after inoculation with inactivated SARS-CoV-2 vaccines [6]. Another study presented two “false-positive” cases of SARS-CoV-2 due to contamination of the specimens with an inactivated virus vaccine strain. The authors suggested that when the syringe needle was pulled out of the vaccine ampoule, the vaccine may have contaminated the cap and outer surface of the bottle [7]. Although there is no risk of infection and transmission with the contamination, it may lead to false-positive clinical test results for COVID-19 [6,8]. Therefore, it is important to develop a method that can determine whether a positive nucleic acid test result is due to contamination from a novel coronavirus inactivated vaccine.

The types of vaccines currently under development are mainly based on DNA, RNA, nonreplicating viral vectors, and inactivated vaccines [9,10], among which the inactivated virus vaccine is obtained by inactivating and purifying SARS-CoV-2 cultured in Vero cells of African green monkey kidney [11]. Trace amounts of Vero cell-derived DNA are known as residual host cell DNA (rcDNA), and qPCR is considered the most practical method for rcDNA quantitation [12]. To date, there is no related kit that combines the nucleic acid target of SARS-CoV-2 and rcDNA of Vero cells to distinguish COVID-19 infection from novel coronavirus inactivated vaccine contamination. Here, we developed a novel one-step reverse transcription real-time PCR (RT-qPCR) targeting the ORF1ab and N genes of SARS-CoV-2 and an alpha-satellite DNA fragment of Vero cells and based on specific primers and hydrolysis probes for these three amplification regions. The diagnostic performance of our RT-qPCR assay was further evaluated to distinguish COVID-19 infection from novel coronavirus inactivated vaccine contamination.

## 2. Materials and Methods

### 2.1. Materials

The verified primers and probes were synthesized by Sangon (Sangon Co., Ltd., Shanghai, China). Armoured RNAs of the ORF1ab and N gene fragments were kindly provided by TopBiotech (TopBiotech Co., Ltd., Xiamen, China), and plasmids containing the Vero cell gene fragment were synthesized by Tsingke (Tsingke Biotechnology Co., Ltd., Beijing, China).

### 2.2. Primer and Probe Design

The nucleic acid sequences of the ORF1ab gene (NC_045512.2:266-21555) and N gene (NC_045512.2:28274-29533) of SARS-CoV-2 and the alpha-satellite sequence of the Vero cell (X04339.1) were obtained from NCBI. NCBI primer-BLAST, DNAMAN, Primer Premier 5, and TM Utility were used for the auxiliary design according to primer design principles. The primers and probes with different fluorescence groups for the RT-qPCR assay are listed in Table 1. Then, genomic and transcriptomic homology comparisons for the designed primers were performed online using the NCBI Primer-BLAST tool to ensure that the designed primers had high specificity and no potential nonspecific amplification targets.

### 2.3. Development and Evaluation of the One-Step RT-qPCR Assay

The one-step RT-qPCR assay (referred to as our RT-qPCR assay) was performed in 96-well plates with a reaction volume of 30 μL. For the assay, 3 μL of 10× Taq Buffer, 0.24 μL of Champagne Taq DNA Polymerase, 0.35 μL of HiScript II Reverse Transcriptase, 0.25 μL of Murine RNase inhibitor, 0.3 μL of heat-labile UDG, 2.4 μL of MgCl2 and 0.6 μL of dNTP mix (Vazyme Biotech Co., Ltd., Nanjing, China), 0.6 μL of forward primers and 0.6 μL of reverse primers for each gene, 0.15 μL of probes for each gene, 13.81 μL of nuclease-free water, and 5 μL of template were mixed per reaction. The cycling conditions for each assay consisted of incubating the template at 55 °C for 5 min and 95 °C for 30 s, followed by 45 cycles of RT-qPCR, with each cycle consisting of 95 °C for 5 s and then 62 °C for 20 s. All reactions were carried out with a SLAN^®^-96S Real-Time PCR System (Hongshi Medical Technology Co., Ltd., Shanghai, China).

#### 2.3.1. Detection of the Amplification Efficiency and Precision of Our RT-qPCR Assay

The RT-qPCR assay amplification efficiency of each gene was evaluated with serially diluted standards. Dilution series were prepared in Tris-EDTA buffer at 3 × 10^7^, 3 × 10^6^, 3 × 10^5^, 3 × 10^4^, and 3 × 10^3^ copies/mL. Three technical replicates were performed at each concentration. The precision of our RT-qPCR assay was determined with an initial concentration of 3 × 10^4^ copies/mL and was expressed as the coefficient of variation (CV), which was expressed as a percentage, across twenty technical replicates.

#### 2.3.2. Lower Limit of Detection (LLOD) of Our RT-qPCR Assay

The assay analytical sensitivity, defined as the lower limit of detection (LLOD) [13,14], was determined for each gene by serially diluting standards from 32 to 1 copy/reaction. Twenty technical replicates were performed for each dilution, and the results were analysed using probit regression. The LLOD, determined through interpolation of the probit curve, was defined as the concentration of input standards in a reaction where the probability of detection was 95%. Then, 20 technical replicates were performed to verify the LLOD.

##### Sample Collection and Ethics Statement

In this study, 346 clinical samples from quarantined hotel personnel, outpatients, and vaccination site staff were collected at Zhongshan Hospital of Xiamen University, School of Medicine, Xiamen University, from 1 January 2022 to 30 April 2022; these samples included 60 clinical SARS-CoV-2-positive samples, 182 randomly selected clinical SARS-CoV-2-negative samples, and 104 vaccination site staff samples. In addition, 132 environmental samples from vaccination sites were collected during the same period. Clinical samples were collected from suspected COVID-19 patients who had undergone nucleic acid tests for SARS-CoV-2. Samples from the vaccination site staff were collected from the persons who worked at vaccination sites. Environmental samples were collected by swabbing all items to which vaccinees and staff may have been exposed at the vaccination sites of Zhongshan Hospital of Xiamen University, such as hand sanitizers, keyboards, pens, and trash cans, and then storing each swab in a sampling tube. All samples were collected from nasopharyngeal swabs or oropharyngeal swabs and tested within four hours or were stored at −80 °C for later use if they could not be tested immediately. This study was approved by the Institutional Ethics Committee of Zhongshan Hospital of Xiamen University, School of Medicine, Xiamen University (No. xmzsyyky2022-168), and was in compliance with national legislation and the Declaration of Helsinki guidelines.

##### Viral Nucleic Acid Purification

Nucleic acids were automatically isolated from 300 μL of the primary sample using a GenePure Pro 96 instrument (Bioer Technology Co., Ltd., Hangzhou, China) and MagaBio Plus VIRUS DNA/RNA purification kit (Bioer Technology Co., Ltd., Hangzhou, China), and then the sample was eluted in 70 μL of elution buffer.

##### Sample Detection and Clinical Management

All samples were concurrently detected in a double-blind manner on the SLAN^®^-96S Real-Time PCR System with our RT-qPCR assay and the Daan SARS-CoV-2 detection kit (referred to as the Daan kit) (Daan Gene Co., Ltd., of Sun Yat-sen University, Guangzhou, China). A Daan kit, which detects the ORF1ab and N genes of SARS-CoV-2, was used according to the manufacturer’s instructions. Standard substances were used as positive controls, and no-template control (NTC) wells were used as negative controls. The Ct cut-off value of the Daan kit and our RT-qPCR assay for the SARS-CoV-2 gene was 35, which is in accordance with the Diagnosis and Treatment of COVID-19 (Trial Version 9) issued by the National Health Commission in 2022 [15].

**Figure 1:**
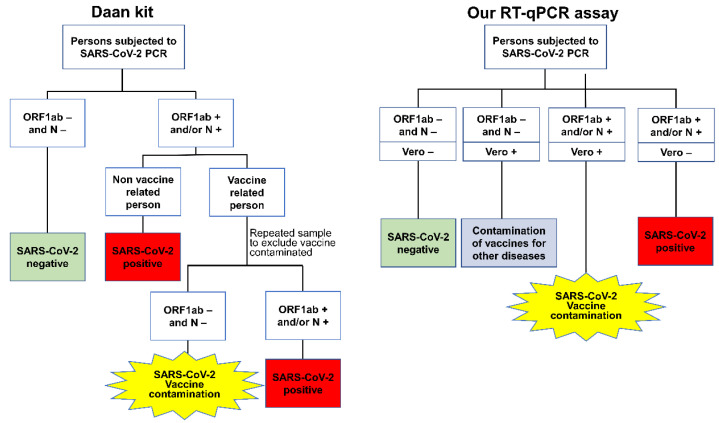
Flow chart of the interpretation of the clinical samples analysed by the Daan kit and our RT-qPCR assay.

The interpretation criteria of the clinical samples (including quarantine hotel personnel, outpatient, and vaccination site staff samples) when using the Daan kit and our RT-qPCR assay are presented as flow charts in Figure 1. For the Daan kit, the definitions were first classified according to the results for the ORF1ab and N genes. Negative test results for both the ORF1ab and N genes were defined as SARS-CoV-2 negative, and the samples that were positive for the ORF1ab and/or N gene were subdivided into two groups according to the vaccine exposure history. The samples from nonvaccine-related persons were defined as SARS-CoV-2 positive based on ORF1ab- and/or N gene-positive results, but the samples from vaccine-related persons were repeatedly sampled to identify the cases of novel coronavirus inactivated vaccine contamination (referred to as SARS-CoV-2 vaccine contamination). Then, repeatedly negative ORF1ab and N gene samples from those who stopped working at the vaccination site for more than 48 h were defined as cases of SARS-CoV-2 vaccine contamination for the primary sample, and repeated positive results for the ORF1ab and/or N gene were defined as SARS-CoV-2-positive.

For our PCR assay, the clinical sample results were defined according to the results of the ORF1ab, N, and Vero genes. SARS-CoV-2-negative samples were defined as samples that were negative for the ORF1ab, N, and Vero genes, and SARS-CoV-2-positive samples were defined as samples that were positive for the ORF1ab and/or N genes and negative for the Vero gene. SARS-CoV-2 vaccine contamination was defined as a sample with positive results for both the SARS-CoV-2 gene (ORF1ab and/or N gene) and Vero gene, while a sample with only Vero gene positivity was defined as contamination of vaccines for other diseases (Figure 1).

For the environmental samples from vaccination site surfaces, samples that were positive for the ORF1ab and/or N gene were defined as SARS-CoV-2 vaccine-contaminated after using the Daan kit; otherwise, the samples were defined as nonvaccine-contaminated. SARS-CoV-2 vaccine contamination was defined as a sample with positive results for both the SARS-CoV-2 gene (ORF1ab and/or N gene) and Vero gene, while a sample with only Vero gene positivity was defined as contamination of vaccines for other diseases; otherwise, the samples were defined as nonvaccine-contaminated. The vaccine involved in this study specifically refers to the inactivated virus vaccine obtained from inactivating and purifying the SARS-CoV-2 virus cultured in Vero cells of African green monkey kidney [9].

### 2.4. Statistical Analysis

According to the above flow chart of the Daan kit and our RT-qPCR assay and the interpretation criteria of the environmental samples, three sets of analyses were performed, and the results of the Daan kit were used as the gold standard to evaluate the diagnostic performance of our assay. For the evaluation of the SARS-CoV-2 diagnostic performance, the samples were classified into a SARS-CoV-2-positive group and a SARS-CoV-2-negative group, the latter including both SARS-CoV-2 vaccine-contaminated samples and SARS-CoV-2-negative samples. For the evaluation of the SARS-CoV-2 vaccine-contamination diagnostic performance, the samples were classified into a SARS-CoV-2 vaccine-contamination group and a nonvaccine-contamination group, the latter including both SARS-CoV-2-positive samples and SARS-CoV-2-negative samples. The environmental samples were also classified into a SARS-CoV-2 vaccine-contamination group and a nonvaccine-contamination group to evaluate the diagnostic performance. The analysis of the diagnostic performance of our RT-qPCR assay included sensitivity, specificity, positive predictive value (PPV), negative predictive value (NPV), positive likelihood ratio (PLR), negative likelihood ratio (NLR), agreement rate, and Kappa value [16]. The statistical analysis was performed using GraphPad Prism software version 8 (GraphPad Software, San Diego, CA, USA) and SPSS 25.0 for Windows (SPSS Inc., Chicago, IL, USA).

## 3. Results

### 3.1. Novel RT-qPCR Amplification Efficiency

**Figure 2:**
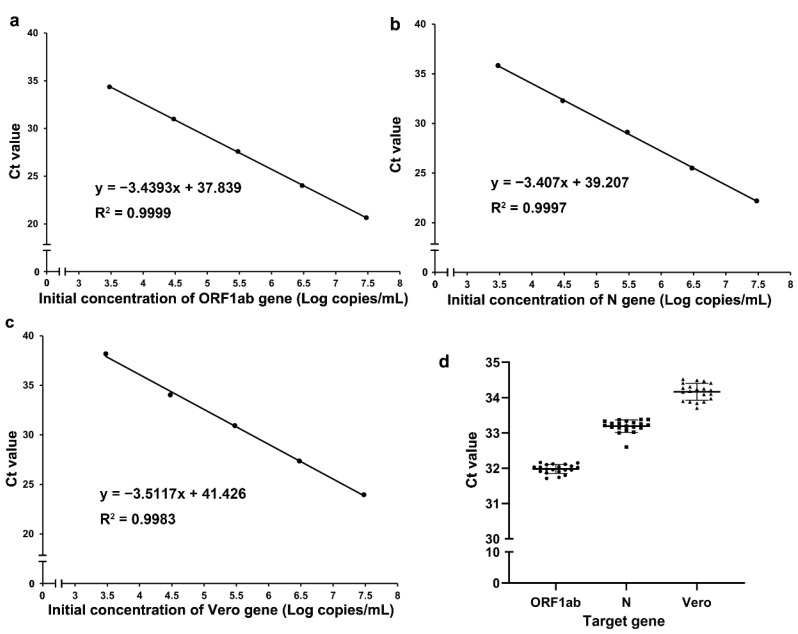
The amplification efficiency and precision of the ORF1ab, N, and Vero genes. (**a**) Analysis of the amplification efficiency of the ORF1ab gene; (**b**) analysis of the amplification efficiency of the N gene; (**c**) analysis of the amplification efficiency of the Vero gene; (**d**) scatter plots based on the Ct values of the ORF1ab, N, and Vero genes.

To analyse the RT-qPCR amplification efficiency, an equimolar mix was used to make a dilution series (for 3 × 10^7^ copies/mL down to 3 × 10^3^ copies/mL), and the Ct value was determined in triplicate using our PCR assay. The results showed that the PCR amplification efficiency of the ORF1ab, N, and Vero genes was 95%, 97%, and 93%, respectively (Figure 2a–c). The linear correlations (R^2^) of the ORF1ab, N, and Vero genes between the Ct value and the copy logarithm were 0.9999, 0.9997, and 0.9983, respectively. These results revealed a high correlation coefficient between the Ct value and the initial concentration of the QRF1ab, N, and Vero genes, and suggested that the novel RT-qPCR had high amplification efficiency.

### 3.2. Novel RT-qPCR Precision

Twenty technical replicates with initial concentrations of 3 × 10^4^ copies/mL were performed to determine the precision of our RT-qPCR assay. All the results showed positive Ct values with coefficients of variation (CVs) < 5% (CV_ORF1ab_ = 0.21%, CV_N_ = 0.16%, and CV_Vero_ = 0.26%). Scatter plots based on the Ct values of the ORF1ab, N, and Vero genes were used to show the distribution of the Ct values (Figure 2d). These results suggested that our RT-qPCR assay had high precision.

### 3.3. Lower Limit of Detection (LLOD) of the Novel RT-qPCR

**Figure 3:**
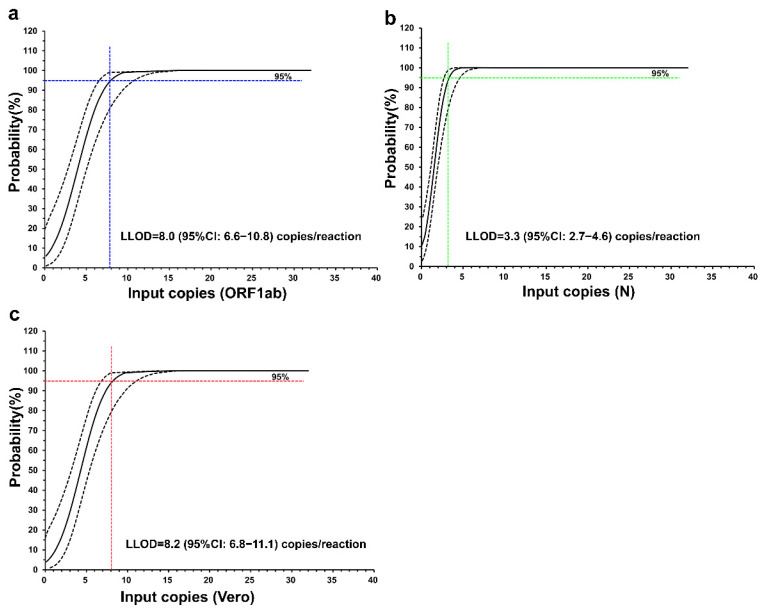
The LLOD of the novel RT-qPCR. (**a**) The probability of detecting the ORF1ab gene (%) at a ratio of 1:2 in serial dilutions of standards from 32 to 1 input copy/reaction using probit regression (solid black line; dashed line denotes the 95% CI). The LLOD, defined as the concentration of the ORF1ab gene in a reaction where the probability of detection in the assay was 95%, was interpolated from the probit curve and is shown as a coloured dashed line; (**b**) the probability of detecting the N gene (%) at a ratio of 1:2 in serial dilutions of standards from 32 to 1 input copy/reaction using probit regression (solid black line; dashed line denotes the 95% CI); (**c**) the probability of detecting the Vero gene (%) at a ratio of 1:2 in serial dilutions of standards from 32 to 1 input copy/reaction using probit regression (solid black line; dashed line denotes the 95% CI).

The LLOD was determined with serially diluted standards (for 32 copies/reaction down to 1 copy/reaction), and the Ct value was determined with our RT-qPCR assay using 20 technical replicates. Probit regression analysis applied to serial dilutions of the synthetic standards revealed that the estimated LLODs of the ORF1ab, N, and Vero genes were 8.0 (95% CI, 6.6–10.8), 3.3 (95% CI, 2.7–4.6), and 8.2 (95% CI, 6.8–11.1) copies per reaction, respectively (Figure 3). Twenty technical replicates were then performed to verify the LLOD, which proved that the detection rate reached 95%.

### 3.4. Evaluation of the SARS-CoV-2 Diagnostic Performance of Our RT-qPCR Assay on Clinical Samples

**Figure 4:**
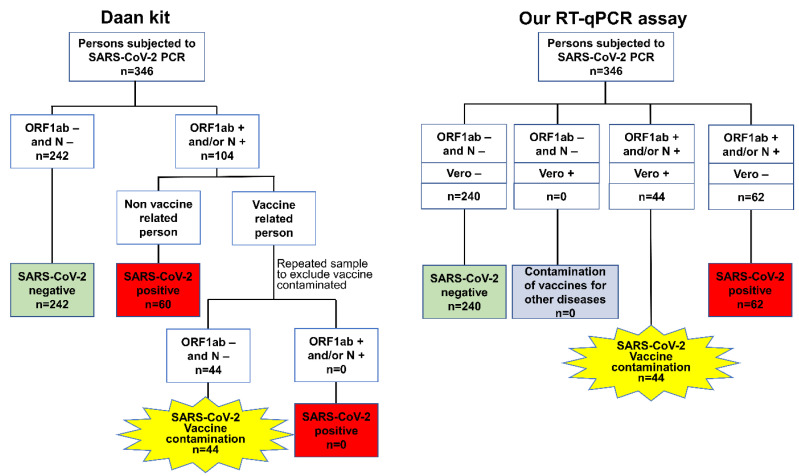
The results of the Daan kit and our RT-qPCR assay according to the interpretation flow chart.

In this study, 346 clinical samples from quarantine hotel personnel, outpatients, and vaccination site staff were collected, and the information is listed in Table 2. The samples were from 140 males (40.5%) and 206 females (59.5%), ranging in age from 8 to 71 years, with an average age of 37.8 years. The number of samples from the quarantine hotel personnel, outpatients, and vaccination site staff was 179 (51.7%), 63 (18.2%), and 104 (30.1%), respectively, with 60 (17.3%) SARS-CoV-2-positive samples and 286 (82.7%) SARS-CoV-2-negative samples. The samples included 179 (51.7%) nasopharyngeal swabs and 167 (48.3%) oropharyngeal swabs. The results of the analyses using the Daan kit and our RT-qPCR assay, which were interpreted through the above flow chart, are presented in Figure 4. The results of the Daan kit were used as the gold standard for evaluating the SARS-CoV-2 diagnostic performance of our RT-qPCR assay (Table 3), in which 60 clinical samples were considered SARS-CoV-2-positive and 286 samples were considered SARS-CoV-2-negative (including SARS-CoV-2-negative samples and SARS-CoV-2 vaccine-contaminated samples). The detection results of our RT-qPCR assay are generally consistent with the Daan kit with a 99.42% agreement (κ value = 0.98 (~0.95–1.01)) for SARS-CoV-2 detection, and the sensitivity, specificity, PLR, and NLR were 100.00%, 99.30%, 143.00, and 0.00, respectively. Our RT-qPCR assay correctly identified 60 SARS-CoV-2-positive samples and 284 SARS-CoV-2-negative samples but misdiagnosed two SARS-CoV-2-negative samples as SARS-CoV-2-positive. None of these SARS-CoV-2-positive samples were identified as positive for the Vero gene. The results indicated that our RT-qPCR assay can correctly diagnose true positive COVID-19 patients.

### 3.5. Evaluation of the SARS-CoV-2 Vaccine-Contamination Diagnostic Performance of Our RT-qPCR Assay on Clinical Samples

In total, 346 clinical samples were tested concurrently in a double-blind manner using the Daan kit and our RT-qPCR assay, and the results showed that 44 samples were considered SARS-CoV-2 vaccine-contaminated and 302 samples were considered nonvaccine-contaminated (including SARS-CoV-2-positive and SARS-CoV-2-negative samples) according to the flow chart for the Daan kit, which was considered the gold standard for evaluating the SARS-CoV-2 vaccine-contamination diagnostic performance of our RT-qPCR assay (Table 4). The detection result of our RT-qPCR assay was consistent with the commercial Daan kit with 100% agreement (κ value = 1) for the vaccination site staff samples, and the sensitivity, specificity, and NLR were 100%, 100%, and 0.00, respectively. The results showed that our RT-qPCR assay correctly identified 44 SARS-CoV-2 vaccine-contaminated samples and 302 nonvaccine-contaminated samples, which indicated that our RT-qPCR assay has the capacity to identify SARS-CoV-2 vaccine-contaminated samples from clinical samples, indicating that our RT-qPCR assay can be used to identify false-positive SARS-CoV-2 test results caused by SARS-CoV-2 vaccine contamination.

### 3.6. Evaluation of the Diagnostic Performance on the Environmental Samples from the Vaccination Site

A total of 132 samples from the vaccination sites were tested in a double-blind manner concurrently using our RT-qPCR assay and Daan kit. According to the interpretation criteria of the environmental samples described above, the results showed that 67 samples were defined as SARS-CoV-2 vaccine-contaminated, and 65 samples were defined as nonvaccine-contaminated using the Daan kit, which was considered the gold standard for evaluating the diagnostic performance of our RT-qPCR assay for the environmental samples from the vaccination site (Table 5). The detection result of our RT-qPCR assay was generally consistent with the commercial Daan kit with a 91.67% agreement (κ value = 0.83 (~0.74–0.93)), and the sensitivity, specificity, PPV, NPV, PLR, and NLR were 88.06%, 95.38%, 95.16%, 88.57%, 19.08, and 0.13, respectively. The results showed that our RT-qPCR assay correctly identified 59 SARS-CoV-2 vaccine-contaminated environmental samples and 62 nonvaccine-contaminated environmental samples but failed to detect eight SARS-CoV-2 vaccine-contaminated samples and misdiagnosed three nonvaccine-contaminated samples as SARS-CoV-2 vaccine-contaminated samples.

## 4. Discussion

COVID-19 represents a great burden and challenge to the global public health system [1]. Nucleic acid testing is the gold standard for the laboratory diagnosis of COVID-19 [2]. The early detection of COVID-19 and the accuracy of results are the basis for timely quarantine measures. Widespread COVID-19 vaccination is considered a strategy for ending the pandemic through herd immunity [5], but aerosol contamination in the vaccination environment may cause false-positive SARS-CoV-2 detection results. At present, identifying false-positive SARS-CoV-2 test results usually involves continuous nucleic acid detection during the quarantine period or sequencing of cases that were suspected to be subject to SARS-CoV-2 vaccine contamination, resulting in a substantial waste of public resources and affecting the normal life of persons who have been misdiagnosed. Therefore, it is of great significance to identify false-positive SARS-CoV-2 test results caused by SARS-CoV-2 vaccine contamination. It is difficult to avoid residual DNA of Vero cells in the purification process of the novel coronavirus inactivated vaccine. qPCR is a recommended method for quantitative rcDNA detection due to its high specificity and sensitivity [12]. There are many SARS-CoV-2 detection kits available on the market, such as the Daan Detection Kit and ZJ Detection Kit, which have achieved high sensitivity in detecting SARS-CoV-2 genes and are widely used in clinical laboratory tests. However, these kits only target the ORF1ab, N, or E genes of SARS-CoV-2. They all have good performance in detecting the SARS-CoV-2 gene but cannot identify false-positive results caused by SARS-CoV-2 vaccine contamination. Misdiagnoses of SARS-CoV-2 vaccine-contaminated persons due to false-positive SARS-CoV-2 test results may cause unnecessary social panic and loss of public resources, but the sequencing of suspected samples is difficult to implement widely. Therefore, the establishment of an assay for the simultaneous detection of SARS-CoV-2 genes and rcDNA derived from Vero cells was the focus of our study. In this study, a one-step multifluorescence RT-qPCR assay was used to detect the ORF1ab, N, and Vero genes. Synthetic armoured SARS-CoV-2 and Vero plasmids were used as standards for evaluation, and the diagnostic performance was verified. The conventional strategy of RNA and DNA profiling entails two separate procedures with different buffer systems. Our RT-qPCR assay is characterized by the simultaneous detection of multiple targets in one reaction, and it is not common for the target genes of a reaction to concurrently included RNA and DNA. The one-step reaction combines the reverse transcription process with the amplification stage, which not only saves time but also greatly reduces laboratory contamination. Our RT-qPCR assay had high amplification efficiency, and the LLOD of the ORF1ab, N, and Vero genes reached 8.0, 3.3, and 8.2 copies per reaction, respectively. The coefficient of variation of the Ct value of all three genes was less than 5%, indicating high precision. When testing clinical samples, our RT-qPCR assay identified SARS-CoV-2-positive and SARS-CoV-2-negative samples with a sensitivity of 100.00% and a specificity of 99.30%, and our RT-qPCR assay accurately identified SARS-CoV-2 vaccine-contaminated and nonvaccine-contaminated samples with a sensitivity of 100% and a specificity of 100%. For the environmental samples, our RT-qPCR assay identified SARS-CoV-2 vaccine-contaminated samples with a sensitivity of 88.06% and a specificity of 95.38%.

Different primer–probe sets have different abilities to detect COVID-19 [17], and different RT-PCR kits for COVID-19 testing have different Ct values and different diagnostic performances [18]. Such differences also existed in our study, resulting in different interpretation results of several samples in the two assays, and this was reflected as samples that were misdiagnosed by our assay. The possible reasons are as follows: first, although in the detection of the SARS-CoV-2 genes, the Daan kit and our RT-qPCR assay both target the ORF1ab and N genes, the sequences of the primers and probes in the two assays were different. Second, the detection results of the high-concentration samples were consistent between the two assays, but the results of the low-concentration samples may be affected by the uneven distributions of the samples. All these results manifested as different amplification efficiencies and different Ct values of the two assays and led to samples whose Ct values were near the critical value presenting different interpretations of the results.

The Ct cut-off value for the SARS-CoV-2 gene was 35 in our study, which is in accordance with the “Diagnosis and Treatment of COVID-19 (Trial Version 9)”. However, samples with low viral loads are characteristic of periods at the beginning and end of infection, presenting a high Ct value. Therefore, when the Ct value of a sample is greater than 35, we suggest that the laboratory provide the specific Ct value to the clinician as a reference given that the samples may have been collected in the early stage or the late stage of the disease and suggest that follow-up and review should be conducted.

The emergence of positive cases of COVID-19 requires immediate and urgent action [19], and false-positive results caused by SARS-CoV-2 vaccine contamination can cause financial and resource losses, unnecessary public isolation, adverse psychological stress, and social panic. When the false-positive results of the vaccination staff caused by SARS-CoV-2 vaccine contamination are considered to be due to viral infections, social panic will result. Therefore, it is important to confirm the physical condition of the vaccination staff. To avoid unnecessary social panic and public resource consumption, regular nucleic acid tests for the environmental surveillance of SARS-CoV-2 are not recommended by the CDC, and no nucleic acid tests should be performed within 48 h of vaccination among persons in contact with the vaccination environment. These measures, which can only eliminate some false-positive results, are cumbersome and have many uncontrollable factors. When false positives are suspected, much work, such as epidemiological investigations of suspected individuals, large-scale environmental monitoring, repeated testing of samples, and sequencing (which is reliable but difficult to apply widely), are often needed. In contrast, our RT-qPCR assay serves as a method that can quickly identify false-positive samples caused by SARS-CoV-2 vaccine contamination, making the physical condition monitoring process for vaccine staff quicker and simpler while preventing unnecessary public resource waste resulting from false-positive reports.

To the best of our knowledge, this is the first study to report an effective and economic strategy for combining SARS-CoV-2 and Vero gene testing to screen for potentially false-positive SARS-CoV-2 test results caused by SARS-CoV-2 vaccine contamination. However, there are limitations in the study that should be considered. First, although the identification capacity of our RT-qPCR assay for vaccine contamination could be confirmed, it is difficult to gain a deeper understanding of the half-life of vaccine nucleic acids in humans to determine appropriate sampling times for specific populations. The second limitation is that the type of vaccine contamination identified in our RT-qPCR assay was inactivated Vero cell-derived virus vaccine contamination. Notably, our RT-qPCR assay cannot identify patients with concurrent SARS-CoV-2 vaccine contamination and novel coronavirus infection. For such special cases, it is important to combine the clinical symptoms and epidemiological history, as well as the vaccination history. False-positive subjects diagnosed with SARS-CoV-2 vaccine contamination still require continuous nucleic acid monitoring and reduced contact with the outside world for a certain period of time until their nucleic acid test results become negative.

## 5. Conclusions

In conclusion, the first one-step RT-qPCR assay, which combines SARS-CoV-2 and Vero gene detection, can be used to diagnose COVID-19 and, to a certain extent, can rule out false-positive results due to SARS-CoV-2 vaccine contamination, thereby preventing social panic, and the waste of public health system and social human resources caused by the misjudgement of positive samples. The RT-qPCR assay can also be used for monitoring environmental vaccine contamination, which is more convincing than using detection kits for SARS-CoV-2 genes individually and more economical and convenient than sequencing samples contaminated with vaccine nucleic acids.

## Figures and Tables

**Table 1:** Primers and probes used for the RT-qPCR assay.

Primer/Probe	Sequences (5′–3′)	Gene Region	Tm ^a^ (°C)	Size (bp)
ORF1ab gene primers and probe				
1ab-FP ^b^	TGACCCTGTGGGTTTTACAC	ORF1ab 226–21,555	60.68	113
1ab-RP ^c^	CATCAGCTGACTGAAGCATGG		62.16	
1ab-Probe	FAM ^d^-CTGCGGTATGTGGAAAGGTT-BHQ1		61.18	
N gene primers and probe				
N-FP	AGCAGTAGGGGAACTTCTCC	N 28,274–29,533	61.50	95
N-RP	CTCTCAAGCTGGTTCAATCTGT		61.08	
N-Probe	HEX ^e^-CTGCTGCTTGACAGATTGAACCA-BHQ1		64.25	
Vero gene primers and probe				
Vero-FP	ATGGTGAACAACGAAATATCTTCC	alpha sat. 1 1–271	60.08	162
Vero-RP	GGACTTCCAAATATAACTTTGCCA		60.02	
Vero-Probe	CY5 ^f^-AGAAGCTTTCTGAGAAACTGCTCTGT-BHQ2		64.97	

^a^ Tm, melting temperature; ^b^ FP, forward primer; ^c^ RP, reverse primer; ^d^ FAM, represents the addition of FAM fluorescence modifications at the 5′ end of the primer; ^e^ HEX, represents the addition of HEX fluorescence modifications at the 5′ end of the primer; ^f^ CY5, represents the addition of CY5 fluorescence modifications at the 5′ end of the primer.

**Table 2:** Clinical data of 346 clinical samples.

Characteristic	No. (*n* = 346)
Sex	
Male	140 (40.5%)
Female	206 (59.5%)
Mean age (years)	37.8 (range, 8–71)
Sample source	
Quarantine hotel	179 (51.7%)
Outpatient	63 (18.2%)
Vaccination site staff	104 (30.1%)
Type of sample	
Nasopharyngeal swab	179 (51.7%)
Oropharyngeal swab	167 (48.3%)
SARS-CoV-2 Diagnostic	
SARS-CoV-2-positive	60 (17.3%)
SARS-CoV-2-negative	286 (82.7%)

**Table 3:** SARS-CoV-2 diagnostic performance of our RT-qPCR assay for 346 clinical samples.

Our RT-qPCR Assay Result	Daan Kit Result (Gold Standard)		Sensitivity (%) (95% CI ^a^)	Specificity (%) (95% CI)	PLR ^b^ (95% CI)	NLR ^c^ (95% CI)	Agreement (%)	κ Value (95% CI)
	Positive	Negative						
Positive	60	2	100.00 (~94.04–100.00)	99.30 (~97.50–99.92)	143.00 (~35.94–569.02)	0.00	99.42	0.98 (~0.95–1.01)
Negative	0	284						

^a^ CI, confidence interval; ^b^ PLR, positive likelihood ratio; ^c^ NLR, negative likelihood ratio.

**Table 4:** SARS-CoV-2 vaccine contamination diagnostic performance of our RT-qPCR assay for 346 clinical samples.

Our RT-qPCR Assay Result	Daan Kit Result (Gold Standard)		Sensitivity (%) (95% CI ^a^)	Specificity (%) (95% CI)	PLR ^b^ (95% CI)	NLR ^c^ (95% CI)	Agreement (%)	κ Value
	Contaminated	Noncontaminated						
Contaminated ^d^	44	0	100.00 (~91.96–100.00)	100.00 (~98.79–100.00)	-	0.00	100.00	1.00
Noncontaminated	0	302						

^a^ CI, confidence interval; ^b^ PLR, positive likelihood ratio; ^c^ NLR, negative likelihood ratio; ^d^ Contaminated, SARS-CoV-2 vaccine-contaminated sample.

**Table 5:** The diagnostic performance of our RT-qPCR assay on 132 environmental samples from vaccination sites.

Our RT-qPCR Assay Result	Daan Kit Result (Gold Standard)		Sensitivity (%) (95%CI ^a^)	Specificity (%) (95%CI)	PPV ^b^ (%) (95%CI)	NPV ^c^ (%) (95%CI)	PLR ^d^ (95%CI)	NLR ^e^ (95%CI)	Agreement (%)	κ Value (95%CI)
	Contaminated	Noncontaminated								
Contaminated ^f^	59	3	88.06 (~77.82–94.70)	95.38 (~87.10–99.04)	95.16 (~86.50–98.99)	88.57 (~78.72–94.93)	19.08 (~6.30–57.82)	0.13 (~0.07–0.24)	91.67	0.83 (~0.74–0.93)
Noncontaminated	8	62								

^a^ CI, confidence interval; ^b^ PPV, positive predictive value; ^c^ NPV, negative predictive value; ^d^ PLR, positive likelihood ratio; ^e^ NLR, negative likelihood ratio; ^f^ Contaminated, SARS-CoV-2 vaccine-contaminated sample.

## Data Availability

The datasets generated during the present study are available from the corresponding author upon reasonable request.
